# Widening access to medicine may improve general practitioner recruitment in deprived and rural communities: survey of GP origins and current place of work

**DOI:** 10.1186/s12909-015-0445-8

**Published:** 2015-10-01

**Authors:** J. Dowell, M. Norbury, K. Steven, B. Guthrie

**Affiliations:** 1 School of Medicine, University of Dundee, Scotland, UK; 2 Raven Song Community Health Centre, Vancouver, BC Canada; 3 Head of Division of Undergraduate Medical Education, School of Medicine Deanery, Ninewells Hospital and Medical School, Level 8, Room LB8 001, Mail box 16, DD1 9SY, Dundee, UK

**Keywords:** Widening access, General Practice, Workforce, Student selection, Manpower, Social class

## Abstract

**Background:**

Widening access to medicine in the UK is a recalcitrant problem of increasing political importance, with associated strong social justice arguments but without clear evidence of impact on service delivery. Evidence from the United States suggests that widening access may enhance care to underserved communities. Additionally, rural origin has been demonstrated to be the factor most strongly associated with rural practice. However the evidence regarding socio-economic and rural background and subsequent practice locations in the UK has not been explored.

The aim of this study was to investigate the association between general practitioners’ (GPs) socio-economic and rural background at application to medical school and demographic characteristics of their current practice.

**Method:**

The study design was a cross-sectional email survey of general practitioners practising in Scotland. Socio-economic status of GPs at application to medical school was assessed using the self-coded National Statistics Socio-Economic Classification. UK postcode at application was used to define urban–rural location. Current practice deprivation and remoteness was measured using NHS Scotland defined measures based on registered patients’ postcodes.

**Results:**

A survey was sent to 2050 Scottish GPs with a valid accessible email address, with 801 (41.5 %) responding. GPs whose parents had semi-routine or routine occupations had 4.3 times the odds of working in a deprived practice compared to those with parents from managerial and professional occupations (95 % CI 1.8–10.2, *p* = 0.001). GPs from remote and rural Scottish backgrounds were more likely to work in remote Scottish practices, as were GPs originating from other UK countries.

**Conclusion:**

This study showed that childhood background is associated with the population GPs subsequently serve, implying that widening access may positively affect service delivery in addition to any social justice rationale. Longitudinal research is needed to explore this association and the impact of widening access on service delivery more broadly.

## Background

Widening Access (WA) in medicine is a subject that is much discussed, subject to great political interest and commonly promoted [1]. Overall WA describes the activity of promoting ‘non-standard’ or ‘socially disadvantaged’ entrants to tertiary education. In the United Kingdom this is typically now focused primarily on socioeconomic disparity. There is ample evidence that the proportion of applicants from underprivileged backgrounds to medicine within the UK remains extremely small and has not changed appreciably over many years, with the highest social class represented some 30 times more frequently than the lowest [2–5].

There appear to be two major drivers of unequal representation of the more socioeconomically deprived in medicine [1]. Firstly, the educational playing field within the United Kingdom is unequal, with widely varying opportunity and achievement as a result. Secondly, many of the less affluent with suitably high academic achievement do not apply to medicine, believing they are not the ‘right material’ or not wishing to join such a ‘posh club’ [6, 7]. Although medical schools in the UK have no specific mandate to address this, there is governmental pressure and some financial incentives to do so [8]. The Selecting for Excellence report published in 2014 has set ten year targets and best practice indicators for medical schools [9]. The report promotes the use of contextual admissions, greater use of multiple mini-interviews (MMIs), aptitude testing and situational judgement tests (SJTs) and outreach programs, all of which have shown effectiveness in widening participation [1, 9]. Specifically targeted foundation courses also offer more promise than graduate entry as a solution but at some cost to both institutions and students [9–11]. This is in keeping with evidence from so-called ‘Pipeline’ programs in the US [7].

There are three key arguments as to why widening access matters - social equity, educational attainment, and health care delivery [12]. The most common argument relates to a desire to moderate inequity within society. In the US this is considered primarily from the perspective of racial disparities, in the UK there is a greater focus on socio-economic disadvantage, though the two are associated in practice. From this perspective, widening access is morally right irrespective of other outcomes.

The second argument concerns the need to craft the best possible doctors, which can be considered in terms of both the educational potential of students and the educational environment of medical schools. To produce the best doctors, medical schools must recruit those with the best potential and not simply the highest prior achievement which is, at least partly, driven by prior educational opportunity. There is now evidence from both general university and medical courses that when matched for school-leaver grades, those from a privileged educational background perform less well at university [13–15]. So-called contextual assessment might help address this, for instance by adjusting offers for school background. The evidence concerning the impact of the educational environment within which students learn is again primarily from the US and suggests an ethnically diverse college population is associated with enhanced learning [12, 16–19]. It is not clear if this is a transferrable finding, for instance if disabled or socio-economically disadvantaged students would correspondingly influence the views of their peers. So widening access socioeconomically may usefully broaden the professional culture but, to our knowledge, this also remains unproven.

Finally, and the prime focus of this study, is the issue of service delivery. One benefit of WA might be to reduce the international challenge of recruiting doctors to serve socioeconomically disadvantaged and rural/remote populations. US evidence suggests a diverse workforce can improve the quality of care, at least for minority populations where ethnic and linguistic diversity may be significant factors [12]. There is evidence that Black and Hispanic primary and secondary care practitioners in the US are more likely to serve poor communities with higher than average proportions of minority residents [20–22]. We are not aware of comparable evidence from Europe or the UK and representativeness per se might not always be advantageous, for instance, if it came at the cost of lower academic standards which could impact on quality of care. It is not clear how transferrable to the UK context these findings may be and the benefits to care from socioeconomic diversity amongst clinicians in the UK or other countries is as yet unproven. Rural origin is the factor most strongly associated with subsequent rural practice with evidence from remote areas of the western world that students recruited from rural backgrounds are more likely to practice in under-served remote and rural areas [23–30]. The World Health Organisation have recommended that targeted admissions policies should be employed in an attempt to increase the number of health professions deciding to work in remote and rural areas [30].

It is again unclear how applicable these findings are to the UK context, since few areas of the UK are very remote [31]. However, there is evidence of difficulty recruiting GPs to socioeconomically deprived and rural areas and that the distribution of GPs does not match need [32, 33]. These concerns are shared worldwide, and a recent literature review highlighted the impact of rurality and isolation on patient safety in the European context [34]. If it could be shown that applicants to medicine from socioeconomically disadvantaged or rural backgrounds in the UK chose to work in more disadvantaged or rural populations, then this would provide evidence that WA could have positive effects on service delivery in terms of the number of GPs practising in these currently under-served areas. There are no published UK studies examining childhood background and where doctors subsequently practise, and since GPs have a much wider choice in where to work with no obligation regarding where they practise for GPs either trained within the UK or abroad, we devised an exploratory study to investigate these effects. This aim of this study was, therefore, to investigate whether socioeconomic background and urban–rural location at the time of entry to medical school was associated with relevant current practice characteristics.

## Methods

The study design was a cross-sectional survey via e-mail. NHS Scotland Information Services Division holds data on all GPs contracted to work in Scottish general practices, but only holds e-mail addresses for a proportion. An email invitation which included a web-link to an online questionnaire was sent in 2010 to the 2,050 (41.8 %) GPs in Scotland for whom email addresses were available. One reminder email was sent during the data collection period. It was not possible to directly compare responders and non-responders, but where possible responders were compared to all Scottish GPs in order to inform a judgement about representativeness. For this purpose, aggregate data on the age and sex of GPs in 2010 were obtained from publicly available sources [35]. Publicly available data on practice location were only available for March 2015 [36].

Outcomes: participants were asked to provide the identifier code for the practice in which they currently worked in order that it could be classified as being either in a socioeconomically deprived or remote setting using NHS Scotland centrally held data. Highly deprived practices were defined as those 80 (7.9 %) Scottish practices where more than 50 % of the registered patient population lived in the most deprived 15 % of Scottish postcodes (a measure used by the Scottish Government to identify and target the most deprived areas). Remote practices were defined as those 191 (18.9 %) Scottish practices where the majority of registered patients live 30 min or more drive time from an urban area of >10,000 people (part of the Scottish Executive Urban–rural Classification [SUERC], also a measure used by Scottish Government for planning purposes) [16].

Explanatory variables: socioeconomic status at the time of application to medical school was assessed using the Office for National Statistics self-coded version of the National Statistics Socio-Economic Classification (NS-SEC) as it applied to their parents or guardians. For Scottish residents, postcode at time of application to medical school was used to define SEURC category. Participants also provided data about their age, sex and year of graduation.

Analysis used PASW Statistics version 17 (IBM). Descriptive analysis used cross-tabulation to examine the distribution of GP characteristics in relation to the two outcomes (working in a remote practice; working in a deprived practice). Binary logistic regression was used to examine univariate and adjusted associations between GP characteristics and the two outcomes. Model fitting was sequential based on the most significantly associated individual variable, with model fit assessed using the Akaike Information Criteria to ensure fitting the most parsimonious model given the small number of outcomes available for evaluation.

The NHS Tayside Research Ethics Service evaluated the study and advised that it did not need full Research Ethics Committee review (reference 10/GA/064).

## Results

One hundred and twenty e-mails were returned as undeliverable or unread, with 801 (response rate 41.5 %) completed questionnaires received between November 2010 and January 2011. Table 1 shows characteristics of responding GPs, where a small majority were male, 61 % were aged 41–55 years, and 47 % graduated in the 1980s. Just over three-quarters of responding GPs had one or more parents in NS-SEC 1 (higher managerial and professional occupations) when they applied to medical school, with only 4.3 % having parents in NS-SEC 5 (semi-routine and routine occupations). There were 69 % of responding GPs living in Scotland at the time they applied to medical school, predominately in urban or accessible (within 30 min’ drive of an urban area) locations. Compared to all Scottish GPs, responders were somewhat younger (61.4 % aged 41–55 vs 48.5 % in Scotland) and slightly more likely to work in a highly deprived practice (8.4 % vs 6.2 %).

**Table 1:** Characteristics of responding GPs

Characteristic	No. ( %, 95 % CI) of responding GPs	No. ( %, 95 % CI) of GPs in Scotland
	*N* = 801^a^	*N* = 4907 for age and sex, *N* = 4863 for current practice^b^
Gender		
Male	413 (51.5, 48.0 to 55.1)	2384 (48.6, 47.2 to 50.0)
Female	383 (47.8, 44.3 to 51.3)	2523 (51.4, 50.0 to 52.8)
Missing	5 (0.6, 2.3 to 15.3)	-
Age		
25–40 years	164 (20.5, 17.8 to 23.5)	1736 (35.4, 34.0 to 36.7)
41–55 years	492 (61.4, 57.9 to 64.8)	2381 (48.5, 47.1 to 49.9)
56–75 years	145 (18.1, 15.5 to 21.0)	790 (16.1, 15.1 to 17.2)
Missing	-	-
Graduation year		
1970–79	180 (22.5, 19.7 to 25.6)	n/a
1980–89	375 (46.8, 43.3 to 50.3)	
1990–99	194 (24.2, 21.3 to 27.4)	
2000–05	49 (6.1, 4.61 to 8.1)	
Missing	3 (3.7, 0.9 to 11.8)	
Socioeconomic status at medical school entry		
NS-SEC 1 (higher managerial/professional)	611 (76.3, 73.2 to 79.2)	n/a
NS-SEC 2 (intermediate occupations)	21 (2.6, 1.7 to 4.1)	
NS-SEC 3 (small employers and self-employed)	56 (7.0, 5.4 to 9.0)	
NS-SEC 4 (lower supervisory and technical)	63 (7.9, 6.1 to 10.0)	
NS-SEC 5 (semi-routine and routine)	34 (4.2, 3.0 to 5.9)	
Missing	16 (2.0, 1.2 to 3.3)	
Urban–rural classification at medical school entry		
Scotland – city	219 (27.3, 24.3 to 30.6)	n/a
Scotland – urban	134 (16.7, 14.3 to 19.5)	
Scotland – accessible	95 (11.9, 9.7 to 14.4)	
Scotland – remote	42 (5.2, 3.9 to 7.1)	
Other UK	183 (22.9, 20.0 to 26.0)	
Non-UK	37 (4.6, 3.3 to 6.4	
Missing	91 (11.4, 9.3 to 13.8)	
Current practice (*n* = 801)		
Remote practice	105 (13.1, 10.9 to 15.7)	643 (13.2, 12.3 to 14.2)
Deprived practice	67 (8.4, 6.6 to 10.6)	301 (6.2, 5.5 to 6.9)

^a^Not all respondents completed all NS-SEC questions, and not all postcodes could be matched to remote/rural lookup files (including non-UK residence at the time of medical school application)

^b^Scottish data for age and sex is for 2010/11 matching the survey date; Scotland data for current practice location is for April 2015 which is the only date for which this data is publicly available

In terms of practice characteristics, 8.4 % of responding GPs worked in a highly deprived practice, compared to 6.2 % of all Scottish GPs (no highly deprived practices were remote in either group). GP gender, age and year of graduation were not significantly associated with working in a deprived practice in either univariate or adjusted analysis (Table 2). Examining respondents with a parent in NS-SEC1, 7.2 % worked in a highly deprived practice compared to 12.7 % of those with a parent in NS-SEC4 (OR 1.87, 95 % CI 0.84 to 4.18) and 23.5 % of those with a parent in NS-SEC5 (OR 3.97, 95 % CI 1.70 to 9.27).

**Table 2:** Characteristics of GPs working in a deprived practice

Characteristic	No (%) working in a deprived practice	Unadjusted OR (95 % CI)	Adjusted OR (95 % CI)	*p*-value (adjusted)
Male (*n* = 413)	36 (8.7)	1	-	
Female (*n* = 383)	31 (8.1)	0.92 (0.56 to 1.52)		
25–40 years (*n* = 164)	11 (6.7)	1	-	
41–55 years (*n* = 492)	48 (9.8)	1.50 (0.76 to 2.97)		
56–75 years (*n* = 145)	8 (5.5)	0.81 (0.32 to 2.08)		
Graduation year				
1970–79 (*n* = 180)	14 (7.8)	1	-	
1980–89 (*n* = 375)	36 (9.6)	1.26 (0.61 to 2.40)		
1990–99 (*n* = 194)	13 (6.7)	0.85 (0.39 to 1.87)		
2000–05 (*n* = 49)	4 (8.2)	1.05 (0.33 to 3.36)		
Socioeconomic status				
NS-SEC 1 (High) (*n* = 611)	44 (7.2)	1	1	
NS-SEC 2 (*n* = 21)	1 (4.8)	0.64 (0.08 to 4.91)	0.64 (0.08 to 4.91)	0.71
NS-SEC 3 (*n* = 56)	4 (7.1)	0.99 (0.34 to 2.87)	0.99 (0.34 to 2.87)	0.94
NS-SEC 4 (*n* = 63)	8 (12.7)	1.87 (0.84 to 4.18)	1.87 (0.84 to 4.18)	0.12
NS-SEC 5 (Low) (*n* = 34)	8 (23.5)	3.97 (1.70 to 9.27)	3.97 (1.70 to 9.27)	0.001

There were 13.1 % of responding GPs working in a remote practice compared to 13.2 % of all Scottish GPs, and this was not associated with GP gender, age and year of graduation in either univariate or adjusted analysis (Table 3). Compared to GPs who lived in a Scottish primary city at the time of application to medical school, those living in accessible (within 30 min’ drive time of an urban area, 23.2 % vs 11.4 %, OR 2.34 95 % CI 1.24 to 4.40) or remote (more than 30 min’ drive time from an urban area, 38.1 % vs 11.4 %, OR 4.78, 95 % CI 2.26 to 10.10) areas at the time of application were more likely to be working in a remote practice. GPs from other UK countries were also more likely to be working in a remote practice (26.8 % vs 11.4 %, OR 2.83, 95 % CI 1.67 to 4.82).

**Table 3:** Characteristics of GPs working in a remote practice

Characteristic	No (%) working in a remote practice	Unadjusted OR (95 % CI)	Adjusted OR (95 % CI)	*p*-value (adjusted)
Male (*n* = 413)	83 (20.1)	1		
Female (*n* = 383)	65 (17.0)	0.81 (0.56 to 1.16)	-	
25–40 years (*n* = 164)	28 (17.1)	1	-	
41–55 years (*n* = 492)	95 (19.3)	1.16 (0.73 to 1.85)		
56–75 years (*n* = 145)	27 (18.6)	1.11 (0.62 to 1.99)		
Graduation year				
1970–79 (*n* = 180)	32 (17.8)	1	-	
1980–89 (*n* = 375)	73 (19.5)	1.12 (0.71 to 1.77)		
1990–99 (*n* = 194)	31 (16.0)	0.88 (0.51 to 1.51)		
2000–05 (*n* = 49)	14 (28.6)	1.85 (0.89 to 3.83)		
Urban–rural classification at medical school entry				
Scotland – city (*n* = 219)	25 (11.4)	1	1	
Scotland – urban (*n* = 134)	9 (6.7)	0.56 (0.25 to 1.24)	0.56 (0.25 to 1.24)	0.16
Scotland – accessible (*n* = 95)	22 (23.2)	2.34 (1.24 to 4.40)	2.34 (1.24 to 4.40)	0.006
Scotland – remote (*n* = 42)	16 (38.1)	4.78 (2.26 to 10.10)	4.78 (2.26 to 10.10)	<0.001
Other UK (*n* = 183)	49 (26.8)	2.83 (1.67 to 4.82)	2.83 (1.67 to 4.82)	<0.001
Non-UK (*n* = 37)	7 (18.9)	1.81 (0.72 to 4.53)	1.81 (0.72 to 4.53)	0.25

## Discussion

This study found that childhood background in terms of parental socioeconomic status and more remote residence was associated with subsequent practice in more deprived and more remote locations respectively. Both relationships appeared to have a dose–response pattern, in that the ‘more rural’ their background or ‘more deprived’ their upbringing was in terms of SES, the more likely they were to work in a setting that mirrored this. However, the only statistically significant effects were seen for the most extreme categories of rurality or SES, although the relatively small size of the study means that confidence intervals for other categories are wide and overlapping. However the proportion of respondents with parents with lower socioeconomic status was small and the majority of GPs working in the most deprived practices still had parents with professional or managerial occupations (reflecting that three-quarters of respondents had such a background). GPs from the other UK countries were also more likely to work in remote practices, possibly explained by doctors moving to Scotland in part because of a preference for rural life. GPs who did not originate from the UK were not more likely than average to work in rural areas. The findings are therefore consistent with the argument that increased contextual selection of students might improve recruitment to under-served communities.

This cross sectional study used retrospective data to estimate socio-economic class and remoteness at the time of application to medical school. It is therefore open to a number of biases, especially given the relatively low response rate and our limited ability to compare responders and non-responders (although responders were similar to all Scottish GPs with the exception of being more likely to be middle-aged, which may reflect that younger GPs are less likely to be partners and therefore less likely to have a permanent e-mail on record). It is also important to note that the area an individual lived or their SES at point of entry to medical school may differ from the experience they had while growing up. However the distribution of respondents’ socioeconomic status at the time they entered medical school is strikingly similar to that of current medical school entrants [3]. Given the limited numbers of doctors either from very deprived or remote areas or working in such areas, some analytic subgroups are small and corresponding confidence intervals wide. Thus, further evidence is clearly needed and long-term prospective studies are ideally required to investigate this more fully as many other factors, such as type of school or medical school attended may also be important, as may recall bias.

The relevant existing literature concerning widening access primarily originates from the USA, Canada and Australia and it is hard to know how transferrable the findings may be to the UK given the differences in health care systems, culture and employment patterns. Our finding that doctors from less affluent backgrounds are more likely to serve highly deprived communities is consistent with US findings [21, 25, 37], and the association between rural background and subsequent remote practice is also consistent with international evidence [23–30]. A recent National Audit Office report highlighted that in 2008, despite an increase in GP numbers of 5,700 over the preceding 10 years, 65 % of Spearhead Primary Care Trusts (areas with poor health outcomes) had fewer GPs per head of population than the national average [33]. This mismatch of need and GP supply indicates that enhancing general practice for deprived communities should clearly be a priority [38], and a key first step in that is improving GP recruitment in these areas to ensure that there are adequate numbers of GPs working in such areas. The literature concerning rural practice should be considered with caution in relation to the UK, as few areas would be considered remote by international standards and the nature of deprivation and resource allocation very localised [39]. We could not locate any relevant peer reviewed literature, suggesting this may also be an area worthy of further study. Research suggests that many GPs remain in the same geographical region as their place of training [40], so considering how training programs can champion practice in rural or deprived areas may be one approach to enhance recruitment and retention. Although our findings may have relevance to recruitment or workforce planning, it is important at an individual level not to presume thatphysicians from a low SES or rural background would wish to, or should be expected to work in a related area.

## Conclusion

This research supports the argument that widening access to medical schools might plausibly improve GP recruitment in for under-served communities. However, if the aim is to improve recruitment of GPs to practices servicing the most deprived or most remote communities, then it is unclear whether this is best achieved by increasing recruitment of medical students from such communities, or ensuring that high-quality undergraduate and postgraduate training opportunities are available to all students and trainees in those areas. In practice, no single ‘magic bullet’ fix is likely to solve recruitment problems in the most deprived and most remote areas, and for many is less important than the promotion of social mobility and social justice. In order to understand whether widening access delivers its intended aims, longitudinal research is needed to understand how academic ability, non-academic skills, demographic and social characteristics are associated with career choice, performance and progression, and eventually quality and safety of care.
